# The Funeral

**Published:** 1898-10

**Authors:** 


### THE FUNERAL.

The funeral of Dr. K. N. Bahadhurji took place on Tuesday morning the 16th August at his residence at Malabar Hill. At an early hour on Tuesday morning, Parsees from all parts of the city were observed going in the direction of Malabar Hill for the purpose of attending the funeral, which was fixed for 8 a. m. The drawing-room, where the body was placed on a marble slab, was occupied by the ladies, and the verandahs and extensive *marquees* erected in the front and rear compounds of the bungalow by gentlemen. By 7 o'clock about a thousand Parsees had congregated, and long before the hour of the funeral several hundreds more arrived, some on foot, and a great many in conveyances, which lined the Ridge down to the end of Gibbs' Road, extending over a mile. Almost the whole of the male adult population of the community attended to pay their last respects to a man who was held in such high esteem during his comparative brief but brilliant professional career. The members of the family of Sir Jamsetjee, the Petits, the Alblesses, the Wadias, the Dadisetts, the Patels, and all the leading families were represented, and there were present no fewer than a hundred members of the medical profession. A large number of grateful patients, both old and young, and a still larger number of students, who had received the benefit of education at the hads of Dr. Bahadhurji when he was a teacher at the Grant Medical College, swelled the attendance. Prayers were said by the priests all night before the body for the repose of the soul of the dead, but the final funeral prayers were recited together by two of the family priests at about 7:30 a. m., and lasted for an hour. At the termination of the last ceremonial the whole assembly rose, and in procession marched past the house and entered by twos and threes into the drawing-room, to have a final look at the face of their deceased friend. The congregation was so large that though the gentlemen walked past the body, which was then placed on an iron bier, at a somewhat rapid pace, it took nearly three-quarters of an hour to accomplish that part of the ceremonial. The mourners then lined the compound on either side, in some places four and five deep, awaiting the passing of the bier. When the bier was conveyed by the corpse-bearers on their shoulders through the lane formed by hundreds of bystanders into the road, the funeral party formed themselves into a procession, the chief mourners being Mr. Nusserwanjee Bahadhurji, the father, and Mr. Dorab Bahadhurji, the younger brother of the deceased. The funeral cortege, which was followed by several hundred Parsees, wended its way from the Ridge down to the Malabar Hill Reservoir, and proceeding to Gibbs' Road turned into the main gate of Towers-of-silence hill. Walking at a slow and measured pace, it took about fifteen minutes to traverse the distrance of about half a mile from the bungalow to the Towers-of-silence, where the funeral party were allowed an opportunity to have a last look of the body before it was consigned to the tower. Prayers were then said for the soul of the dead, and the mourners both at the house and the towers dispersed.—*The Indian Lancet.*

